# Second-line treatment for primary central nervous system lymphoma

**DOI:** 10.1038/sj.bjc.6690083

**Published:** 1999-02

**Authors:** M Reni, A J M Ferreri, E Villa

**Affiliations:** Department of Radiochemotherapy, San Raffaele H Scientific Institute, via Olgettina 60, Milan, 20132, Italy

**Keywords:** primary central nervous system lymphoma, salvage treatment, brain neoplasms, extranodal lymphomas, non-Hodgkin's lymphoma

## Abstract

Failure after first-line treatment was reported in 35–60% of immunocompetent patients with primary central nervous system lymphoma (PCNSL). There are currently no reports focusing on salvage therapy. This review analyses prognostic factors and the efficacy of salvage therapy by focusing on data from papers reporting results of first-line treatment in 355 cases. The study group consisted of 173 patients presenting treatment failure. The interval between failure and death (TTD) was compared for age at relapse (≤60 vs >60 years), type of failure (relapse vs progression), time to relapse (≤12 vs >12 months) and salvage treatment (yes vs no). Median TTD was similar in younger and older patients (*P* = 0.09). Relapsed patients had a longer TTD than patients with progressive disease (*P* = 0.002). Early relapse led to a shorter TTD than late relapse (*P* = 0.005). Median TTD was 14 months for patients who underwent salvage therapy and 2 months for untreated cases (*P* < 0.00001). A multivariate analysis showed an independent prognostic role for salvage therapy and time to relapse. Age and type of failure had no predictive value. Salvage therapy significantly improves outcome and, possibly, quality of life. As many different treatments were used conclusions cannot be made regarding an optimal treatment schedule. © 1999 Cancer Research Campaign


*British Journal of Cancer* (1999) **79**(3/4), 530–534
© 1999 Cancer Research Campaign
Article no. bjoc.1998.0083

### Second-line treatment for primary central nervous system lymphoma

**M Reni, AJM Ferreri and E Villa**

Department of Radiochemotherapy, San Raffaele H Scientific Institute, via Olgettina 60, 20132 Milan, Italy

**Summary** Failure after first-line treatment was reported in 35–60% of immunocompetent patients with primary central nervous system lymphoma (PCNSL). There are currently no reports focusing on salvage therapy. This review analyses prognostic factors and the efficacy of salvage therapy by focusing on data from papers reporting results of first-line treatment in 355 cases. The study group consisted of 173 patients presenting treatment failure. The interval between failure and death (TTD) was compared for age at relapse (≤60 vs >60 years), type of failure (relapse vs progression), time to relapse (≤12 vs >12 months) and salvage treatment (yes vs no). Median TTD was similar in younger and older patients ($P = 0.09$). Relapsed patients had a longer TTD than patients with progressive disease ($P = 0.002$). Early relapse led to a shorter TTD than late relapse ($P = 0.005$). Median TTD was 14 months for patients who underwent salvage therapy and 2 months for untreated cases ($P < 0.00001$). A multivariate analysis showed an independent prognostic role for salvage therapy and time to relapse. Age and type of failure had no predictive value. Salvage therapy significantly improves outcome and, possibly, quality of life. As many different treatments were used conclusions cannot be made regarding an optimal treatment schedule.

**Keywords:** primary central nervous system lymphoma; salvage treatment; brain neoplasms; extranodal lymphomas; non-Hodgkin's lymphoma

Because of the growing interest over the past decade in primary central nervous system lymphoma (PCNSL), more information is now available on optimal primary treatment, which has made a significant impact on survival. Recent prospective series (De Angelis et al, 1992; Glass et al, 1994, 1996) and review studies (Jellinger and Paulus, 1992; Fine and Mayer, 1993; Ferreri et al, 1995*a*; Reni et al, 1997) have shown that the combination of chemotherapy (CHT) containing high-dose methotrexate and radiotherapy (RT), delivering more than 40 Gy to the whole brain, plus a boost that exceeds 50 Gy to the tumour bed doubles survival compared with RT alone. Despite the high complete remission rate achieved with first-line therapy, 35–60% of patients submitted to combined treatment modality (CTM) relapse and die of lymphoma within a few months from recurrence (De Angelis et al, 1992; Glass et al, 1994; Selch et al, 1994; Sarazin et al, 1995). Most patients presenting progressive or relapsed disease did not receive additional treatment and, unlike extraneural non-Hodgkin's lymphoma (NHL), the impact of salvage therapy on survival and quality of life remains unclear because of the lack of reports focusing on this issue. The present paper analyses prognostic factors and the impact on survival of second-line therapy in PCNSL by reviewing published series reporting the therapeutic results for first-line treatment.

### MATERIALS AND METHODS

All papers on immunocompetent patients with PCNSL published in English literature were considered. Complete data of disease-free survival (DFS), overall survival (OS) and management of recurrent or progressive disease after first-line treatment were available for each patient in 24 out of 59 considered series (Sagerman et al, 1967; Rampen et al, 1980; Letendre et al, 1982; Gonzalez and Schuster-Uitterhoeve, 1983; Mendenhall et al, 1983; Loeffler et al, 1985; Woodman et al, 1985; Di Marco et al, 1986; Murray et al, 1986; Vakili et al, 1986; Yasunaga et al, 1986; McLaughlin et al, 1988; Shibamoto et al, 1990; Uematsu et al, 1992; Watne et al, 1992; Boiardi et al, 1993; Glass et al, 1994, 1996; Lachance et al, 1994; Ling et al, 1994; Selch et al, 1994; Ferreri et al, 1995*b*; Sarazin et al, 1995; Freilich et al, 1996). Three hundred and fifty-five cases were reported in the analysed series: 334 patients received RT, CHT or CTM as first-line treatment, whereas 21 patients were not submitted to any therapy after diagnosis. The present review focuses on the 173 patients presenting recurrent or progressive disease after first-line therapy, whereas 161 were excluded from analysis because they were either relapse-free, lost to follow-up, dead without evidence of disease or dead of treatment toxicity at the time of report.

Age at failure, time to relapse and type of failure are summarized in Table 1. Age was reported in 160 cases: 91 (56.8%) were ≤60 years old and 69 (43.2%) >60 years. Performance status at treatment failure was never available. Failure consisted of relapse after an initial objective response in 120 patients, and progressive disease in 53 patients. The interval between primary diagnosis and relapse was defined as time to relapse (TTR), whereas the interval between failure and death was defined as time to death (TTD). Relapse was considered 'early' if it occurred at 12 months or less ($n = 68$) and 'late' if it occurred thereafter ($n = 52$). Fifty-nine cases were submitted to salvage therapy at time of relapse ($n = 52$) or progression ($n = 7$), whereas 114 received no treatment at time of relapse ($n = 68$) or progression ($n = 46$). Second-line treatment varied; 26 different therapeutic schedules were used in 55 cases,

*Received 25 February 1998*
*Revised 29 May 1998*
*Accepted 9 June 1998*

*Correspondence to:* M Reni, Department of Radiochemotherapy, San Raffaele Scientific Institute, via Olgettina 60, 20132 Milan, Italy

**Table 1** Age, time to relapse (TTR) and type of failure for patients submitted or not to salvage therapy

| Retreated patients | n | Type of failure | | TTR (months) | | Age (years) | |
|---|---|---|---|---|---|---|---|
| | | Rel | PD | <12 | ≥12 | ≤60 | >60 |
| Yes | 59 | 52 | 7 | 29 | 23 | 36 | 15 |
| No | 114 | 68 | 46 | 39 | 29 | 55 | 54 |

Rel, relapse; PD, progressive disease.

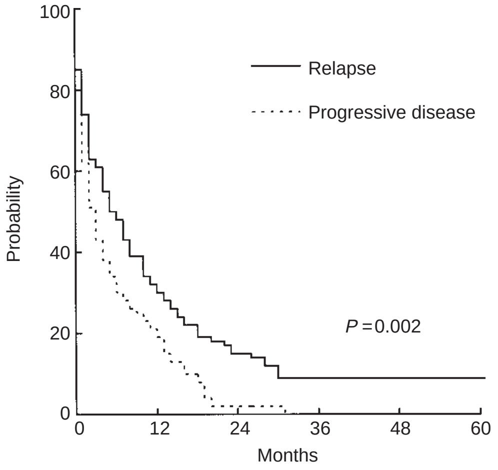

**Figure 1** Time to death curves according to the type of failure after first-line treatment whereas the CHT regimen was not reported in the remaining four patients. At time of failure, 36 patients were treated with RT alone ($n = 24$) or in combination with CHT ($n = 12$), whereas 23 patients underwent CHT as exclusive salvage treatment.

TTR and TTD curves were generated using the Kaplan–Meier method (Kaplan and Meier, 1958). Impact on TTD of age (≤60 years vs >60 years), type of failure (recurrent vs progressive disease), TTR (early vs late relapse), second-line treatment (yes vs no) and use of RT (yes vs no) was evaluated by comparing the TTD curves by the log-rank test (Peto and Pike, 1973). A multivariate analysis by the Cox proportional hazard model (Cox, 1972) was performed on the entire patient group to evaluate the independent role of age, type of failure and retreatment. This analysis, however, did not assess the predictive value of TTR due to the inclusion of patients with progressive disease. A multivariate analysis on TTR, age and salvage treatment, limited to relapsed patients, was also performed.

### RESULTS

The predictive value of age on TTD was analysed. Median TTD was 6 months for patients ≤60 years and 3 months for >60 years ($P = 0.09$).

TTD of 120 patientss with relapsing disease was compared with TTD of 53 patients with progressive disease. The median age in the two groups overlapped (59 and 58 years respectively). Median TTD was 5 and 3 months respectively ($P = 0.002$) (Figure 1). Median TTR for the entire group of 120 relapsed patients was 10 months.

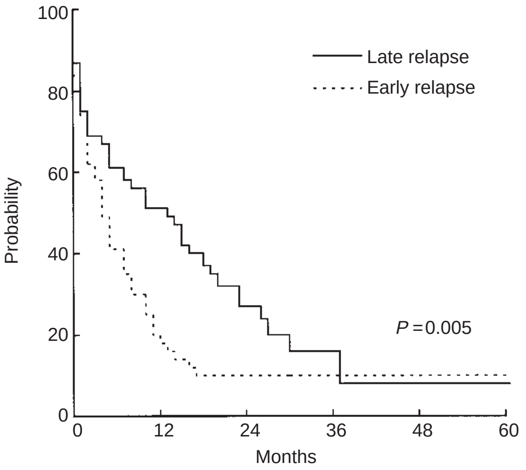

**Figure 2** Time to death curves according to the duration of time to relapse after first-line treatment

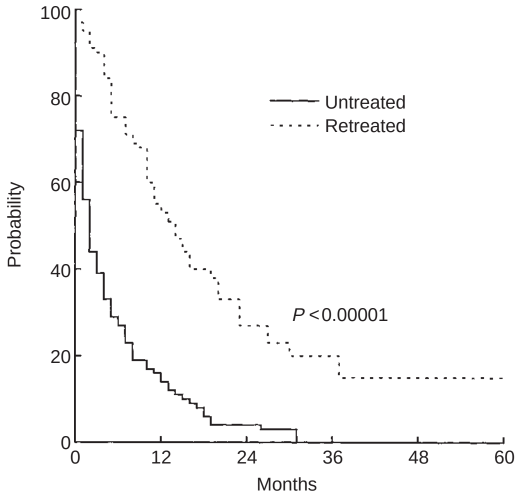

**Figure 3** Comparison of time to death curves for retreated (……) or untreated (—) patients

Early relapse occurred in 68 patients, with a median age of 60 years (range 1–89) and a median TTD of 4 months. Fifty-two patients had late relapse. Their median age was 55 years (range 14–76), and the median TTD was 10 months. The difference between TTD curves was statistically significant ($P = 0.005$) (Figure 2).

The median age of patients receiving and of those not receiving second-line therapy was 55 and 59 years respectively. Median TTD for patients submitted to salvage therapy ($n = 59$) and untreated patients ($n = 114$) was 14 and 2 months respectively ($P < 0.00001$) (Figure 3). For retreated patients, 1- and 3-year actuarial TTD were 53% and 15%. Two patients were still alive 5 years after failure. Meanwhile, 1- and 3-year actuarial TTD was 14% and 0% for untreated patients.

Salvage therapy significantly improved survival in each patient subset based on age groups, on time to relapse or on type of failure ($P < 0.001$ in each analysis). Inclusion of RT in second-line treatment schedules, either in combination with CHT or alone, raised the mTTD to 16.5 months compared with the 10 months obtained with chemotherapy alone ($P = 0.03$) (Figure 4).

© Cancer Research Campaign 1999

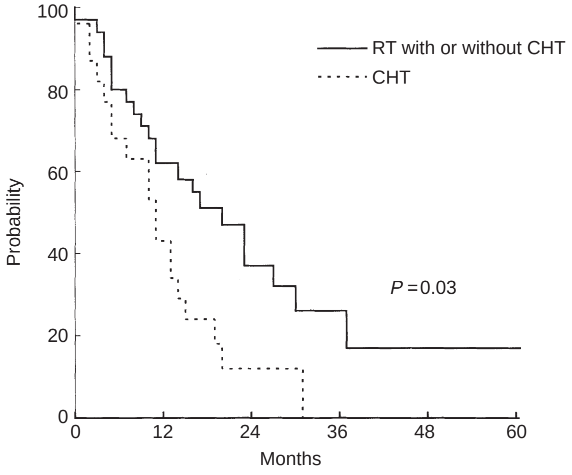

**Figure 4** Comparison of time to death curves for patients receiving RT with or without CHT (—) or CHT alone (······) as second-line treatment

Results of multivariate analyses by the Cox proportional hazard model performed on the entire study group ($n$ = 160) and on relapsed patients ($n$ = 110) to evaluate age, TTR, type of failure and salvage therapy showed that only salvage therapy and TTR had prognostic value (relative risk = 3.5 and 0.57; $P$ = 0.000001 and 0.01 respectively).

### DISCUSSION

Relapse of extraneural NHL had been considered fatal until the advent of high-dose chemotherapy followed by autologous or allogenic bone marrow or peripheral blood stem cell transplantation. However, only a subset of relapsed patients can be selected for such life-threatening treatment, whereas most patients undergo standard salvage chemotherapy, achieving an overall response rate of 45–85%, with 20–45% complete response (CR), a median survival of 8–14 months and a 3-year survival rate of 20–30% (Velasquez et al, 1994; Rodriguez et al, 1995). About one-half of immunocompetent patients with PCNSL, achieving CR with primary treatment, relapse, whereas 10–15% present refractory disease (De Angelis et al, 1992; Glass et al, 1996). Furthermore, half of the 5-year survivors relapse after 5–13 years from diagnosis (Murray et al, 1986). Prognosis for both recurrent and progressive PCNSL is poor, and most patients die within 2–4 months because of neurological deterioration (Pollack et al, 1989; De Angelis et al, 1992). Brain relapse is the overwhelming cause of failure in PCNSL. The disease, which is responsible for progressive neurological deficits and a poor quality of life, is invariably fatal. The role of salvage therapy for PCNSL has not yet been defined because there is no report on this issue, and more than half of the papers on first-line treatment do not report data on TTR, overall survival and management of recurrent or refractory patients. However, some anecdotal observations have suggested that second-line treatment could achieve a further remission and consequent symptomatic improvement. Neuwelt et al (1991) retreated nine out of ten recurrent PCNSL: four were still alive and disease-free at time of report. Pollack et al (1989) observed a statistically significant survival improvement in ten patients receiving second-line therapy compared with five untreated cases ($P$ < 0.05). Four retreated patients were still alive 6–48 months after recurrence. In contrast, a few authors reported poor results with salvage therapy (Sociè et al, 1990; Nelson et al, 1992; Krogh-Jensen et al, 1994). However, the number of patients was small and data not complete.

This review attempted to identify the prognostic value of some variables such as age at relapse, time to relapse and type of failure, and the impact on survival of salvage treatment at recurrence or progression. Both univariate and multivariate analyses showed that, unlike PCNSL at first diagnosis, age at failure had no predictive value on survival, and that early relapse led to a worse outcome than late relapse.

It is difficult to interpret the results of the analysis of type of failure as a prognostic factor. Univariate analysis showed that patients with recurrent PCNSL survived significantly longer than those with refractory disease. However, this result could be biased because of the prevalence of untreated patients in the group with progressive disease (46 out of 53), whereas 52 out of 120 relapsed patients were retreated. Alternatively, multivariate analysis did not show any difference in relative risk between recurrent and progressive disease. This unexpected result could depend on a diverse definition of refractory and recurrent disease in the papers considered, with this diversity probably leading to the inclusion of some patients with progressive disease in the relapse group.

Salvage therapy was the variable with the highest predictive value of TTD. The significant improvement of outcome in retreated patients was evident in both univariate and multivariate analyses. However, the lack of performance status (PS) data could constitute a bias because patients with lower PS are rarely proposed for salvage therapy. In an attempt to exclude this bias, the comparison between TTD curves of retreated and untreated patients was also carried out by excluding, only in the latter group, patients with TTD ≤2 months, who probably had worse PS. The log-rank test again showed a statistically significant difference between curves ($P$ = 0.01, data not shown).

The present review suggests the efficacy of salvage therapy in terms of survival improvement for patients grouped on the basis of type of failure, TTR and age. Outcome of salvage therapy was probably underestimated because of the inclusion, in the retreated group, of patients receiving ineffective CHT regimens. The high rate of second CR probably had an impact on the retreated patients' quality of life, even though no author studied this issue thoroughly. Because of the many different types of first- and second-line treatment used in the analysed papers, no conclusion could be drawn concerning optimal schedule. Cyclophosphamide, doxorubicin, vincristine and prednisone (CHOP) regimen at recurrence obtained poorer results than drugs that permeate the blood–brain barrier (BBB) (De Angelis et al, 1992). In the papers considered, all three patients receiving CHOP at failure died within 8 months. Also as first-line therapy, CHOP or cyclophosphamide, doxorubicin, vincristine and dexamethasone (CHOD) regimens were ineffective (Lachance et al, 1994; O'Neill et al, 1995; Bessell et al, 1996; Glass et al, 1996; Schultz et al, 1996). A salvage treatment schedule should include drugs that can cross the BBB and, in particular, agents with some efficacy as primary treatment, such as high-dose methotrexate, cytarabine, vincristine, procarbazine or nitrosureas (Kawakami et al, 1985; Di Marco et al, 1986; McLaughlin et al, 1988; Chamberlain and Levin, 1990; De Angelis et al, 1992; Boiardi et al, 1993; Glass et al, 1994; Bessel et al, 1996; Freilich et al, 1996; Reni et al, 1997). In the current review, median survival and TTD were better in patients receiving irradiation with or without CHT at failure than in patients who underwent CHT alone. Twenty-five out of 36 cases in the first group had been previously irradiated. The heterogeneity

© Cancer Research Campaign 1999

of both first- and second-line RT doses and fields does not allow us to give precise indications. However, RT most probably has a role in salvage treatment. To better define optimal management of relapsing or progressive PCNSL, reporting of single-centre experience should be encouraged. Furthermore, the description of second-line therapy in series of primary treatment of PCNSL appears mandatory because of the statistically significant impact on outcome.

In conclusion, salvage therapy in recurrent or progressive PCNSL appears worthwhile because it achieves an increment of TTD compared with untreated patients. It also apparently reduces the intensity of neurological symptoms related to failure, thus improving the quality of life. Further improvement of outcome in PCNSL could derive from a better definition of an optimal treatment schedule for both the first- and the second-line therapy.

### REFERENCES

Bessell EM, Graus F, Punt JAG, Firth JL, Hope T, Moloney AJ, Lopez-Guillermo A and Villa S (1996) Primary non-Hodgkin's lymphoma of the CNS treated with BVAM or CHOD/BVAM chemotherapy before radiotherapy. *J Clin Oncol* **14**: 945–954

Boiardi A, Silvani A, Valentini S, Salmaggi A, Allegranza A and Broggi G (1993) Chemotherapy as first treatment for primary malignant non-Hodgkin's lymphoma of the central nervous system preliminary data. *J Neurol* **241**: 96–100

Chamberlain MC and Levin VA (1990) Adjuvant chemotherapy for primary lymphoma of the central nervous system. *Arch Neurol* **47**: 1113–1116

Cox DR (1972) Regression models and life-tables. *J R Stat Soc* **34**: 187–220

De Angelis L, Yahalom J, Thaler HT and Kher U (1992) Combined modality therapy for primary CNS lymphoma. *J Clin Oncol* **10**: 635–643

Di Marco A, Rosta L, Campostrini F, Bonetti A, Palazzi M and Garusi G (1986) The role of radiation therapy in the management of primary non-Hodgkin's lymphomas of the central nervous system: clinical study of 10 cases. *Tumori* **72**: 565–573

Ferreri AJM, Reni M and Villa E (1995*a*) Primary central nervous system lymphoma in immunocompetent patients. *Cancer Treat Rev* **21**: 415–446

Ferreri AJM, Reni M, Bolognesi A, Verusio C and Villa E (1995*b*) Combined therapy for primary central nervous system lymphoma in immunocompetent patients. *Eur J Cancer* **31A**: 2008–2012

Fine HA and Mayer RJ (1993) Primary central nervous system lymphoma. *Ann Intern Med* **119**: 1093–1104

Freilich RJ, Delattre JY, Monjour A and De Angelis LM (1996) Chemotherapy without radiation therapy as initial treatment for primary CNS lymphoma in older patients. *Neurology* **46**: 435–439

Glass J, Gruber ML, Cher L and Hochberg FH (1994) Pre-irradiation methotrexate chemotherapy of primary central nervous system lymphoma: long-term outcome. *J Neurosurg* **81**: 188–195

Glass J, Shustik C, Hochberg FH, Cher L and Gruber ML (1996) Therapy of primary central nervous system lymphoma with pre-irradiation methotrexate, cyclophosphamide, doxorubicin, vincristine and dexamethasone (MCHOD). *J Neurooncol* **30**: 257–265

Gonzalez D and Schuster-Uitterhoeve LJ (1983) Primary non-Hodgkin's lymphoma of the central nervous system. Results of radiotherapy in 15 cases. *Cancer* **51**: 2048–2052

Jellinger KA and Paulus W (1992) Primary central nervous system lymphoma – an update. *J Cancer Res Clin Oncol* **119**: 7–27

Kaplan EL and Meier P (1958) Non-parametric estimations from incomplete observations. *J Am Stat Assoc* **53**: 457–481

Kawakami Y, Tabuchi K, Ohnishi K, Asari S and Nishimoto A (1985) Primary central nervous system lymphoma. *J Neurosurg* **62**: 522–527

Krogh-Jensen M, d'Amore F, Jensen MK, Christensen BE, Thorling K, Pedersen M, Johansen P, Boesen AM and Andersen E (1994) Incidence, clinicopathological features and outcome of primary central nervous system lymphomas. *Ann Oncol* **5**: 349–354

Lachance DH, Brizel DM, Gockerman JP, Halperin EC, Burger PC, Boyko OB, Brown MT and Schold Jr SC (1994) Cyclophosphamide, doxorubicin, vincristine, and prednisone for primary central nervous system lymphoma: short duration response and multifocal intracerebral recurrence preceding radiotherapy. *Neurology* **44**: 1721–1727

Letendre L, Banks PM, Reese DF, Miller RH, Scanlon PW and Kiely JM (1982) Primary lymphoma of the central nervous system. *Cancer* **49**: 939–943

Ling SM, Roach M, Larson DA and Wara WM (1994) Radiotherapy of primary central nervous system lymphoma in patients with and without human immunodeficiency virus. Ten years of treatment experience at the university of California san Francisco. *Cancer* **73**: 2570–2582

Loeffler JS, Ervin TJ, Mauch P, Skarin A, Weinstein HJ, Canellos G and Cassady JR (1985) Primary lymphomas of the central nervous system: patterns of failure and factors that influence survival. *J Clin Oncol* **3**: 490–494

McLaughlin, Velasquez WS, Redman JR, Yung WKA, Hagermeister FB, Rodriguez MA and Cabanillas F (1988) Chemotherapy with dexamethasone, high-dose cytarabine, and cisplatin for parenchymal brain lymphoma. *J Natl Cancer Inst* **80**: 1408–1412

Mendenhall NP, Thar TL, Agee OF, Harty-Golder B, Ballinger Jr WE and Million RR (1983) Primary lymphoma of the central nervous system: computerized tomography scan characteristics and treatment results for 12 cases. *Cancer* **52**: 1993–2000

Murray K, Kun L and Cox J (1986) Primary malignant lymphoma of the central nervous system. *J Neurosurg* **65**: 600–607

Nelson DF, Martz KL, Bonner H, Nelson JS, Newall J, Kerman HD, Thomson JW and Murray KJ (1992) Non-Hodgkin's lymphomas of the brain: can high dose, large volume radiation therapy improve survival? Report on a prospective trial by the radiation therapy oncology group (RTOG): RTOG 8315. *Int J Radiat Oncol Biol Phys* **23**: 9–17

Neuwelt EA, Goldman DL, Dahlborg SA, Crossen J, Ramsey F, Roman-Goldstein S, Braziel R and Dana B (1991) Primary CNS lymphoma treated with osmotic blood–brain barrier disruption: prolonged survival and preservation of cognitive function. *J Clin Oncol* **9**: 1580–1590

O'Neill BP, O'Fallon JR, Earle JD, Colgan JP, Brown LD and Krigel RL (1995) Primary central nervous system non-Hodgkin's lymphoma: survival advantages with combined initial therapy? *Int J Radiat Oncol Biol Phys* **33**: 663–673

Peto R and Pike MC (1973) Conservatism of the approximation (O–E)/E in the LogRank test for survival data or tumor incidence data. *Biometrics* **29**: 579–583

Pollack IF, Dade Lunsford L, Flickinger JC and Dameshek HL (1989) Prognostic factors in the diagnosis and treatment of primary central nervous system lymphoma. *Cancer* **63**: 939–947

Rampen FHJ, van Andel JG, Sizoo W and van Unnik JAM (1980) Radiation therapy in primary non-Hodgkin's lymphomas of the CNS. *Eur J Cancer* **16**: 177–184

Reni M, Ferreri AJM, Garancini MP and Villa E (1997) Therapeutic management of primary central nervous system lymphoma in immunocompetent patients: results of a critical review of the literature. *Ann Oncol* **8**: 227–234

Rodriguez MA, Cabanillas FC, Hagemeister FB, McLaughlin P, Romaguera J, Swan F and Velasquez WS (1995) A phase II trial of mesna/ifosfamide, mitoxantrone and etoposide for refractory lymphomas. *Ann Oncol* **6**: 609–611

Sagerman RH, Cassady JR and Chang CH (1967) Radiation therapy for intracranial lymphoma. *Radiology* **88**: 552–554

Sarazin M, Ameri A, Monjour A, Nibio A, Poisson M and Delattre JY (1995) Primary central nervous system lymphoma: treatment with chemotherapy and radiotherapy. *Eur J Cancer* **31A**: 2003–2007

Schultz C, Scott C, Sherman W, Donahue B, Fields J, Murray K, Fisher B, Abrams R and Meis-Kindblom J (1996) Preirradiation chemotherapy with cyclophosphamide, doxorubicin, vincristine and dexamethasone for primary CNS lymphomas: initial report of Radiation Therapy Oncology Group Protocol 88-06. *J Clin Oncol* **14**: 556–564

Selch MT, Shimizu KT, De Salles AF, Sutton C and Parker RG (1994) Primary central nervous system lymphoma. *Am J Clin Oncol* **17**: 286–293

Shibamoto Y, Tsutsui K, Dodo Y, Yamabe H, Shima N and Abe M (1990) Improved survival rate in primary intracranial lymphoma treated by high-dose radiation and systemic Vincristine–Doxorubicin–Cyclophosphamide–Prednisolone chemotherapy. *Cancer* **65**: 1907–1912

Soclé G, Piprot-Chauffat C, Schlienger M, Legars D, Thurel C, Mikol J, Ifran N, Briere J, Pene F, Gindrey-Vie B, Marin JL, Desablens B and Laugier A (1990) Primary lymphoma of the central nervous system: an unresolved therapeutic problem. *Cancer* **65**: 322–326

Uematsu M, Kondo M, Dokiya T, Oguchi Y, Toya S, Torikata C, Kuribayashi T and Hashimoto S (1992) Primary non-AIDS related brain lymphoma. Patterns of failure following radiotherapy. *Acta Oncol* **31**: 551–554

Vakili ST, Muller J, Shidnia H and Campbell RL (1986) Primary lymphoma of the central nervous system: a clinicopathologic analysis of 206 cases. *J Surg Oncol* **33**: 95–102

Velasquez WS, McLaughlin P, Tucker S, Hagemeister FB, Swan F, Rodriguez MA, Romaguera J, Rubenstein E and Cabanillas F (1994) ESHAP – an effective chemotherapy regimen in refractory and relapsing lymphoma: a 4-year follow-up study. *J Clin Oncol* **12**: 1169–1176

Watne K, Scott H, Hager B, Lindegaard MW, Nome O, Abrahamsen AF and Hirschberg H (1992) Primary malignant lymphoma of the brain. A report of 24 cases from the Norwegian Radium Hospital. *Acta Oncol* **31**: 545–550

Woodman R, Shin K and Pineo G (1985) Primary non-Hodgkin lymphoma of the brain: a review. *Medicine* **64**: 425–430

Yasunaga T, Takahashi M, Uozumi H, Takada C, Kawano S, Baba Y, Nakamura I, Sonoda H and Matsukado Y (1986) Radiation therapy of primary malignant lymphoma of the brain. *Acta Radiol Oncol* **25**: 23–28

*© Cancer Research Campaign 1999*
